# Supplementary material for: Fusion of the Cas9 endonuclease and the VirD2 relaxase facilitates homology-directed repair for precise genome engineering in rice
Source: Commun Biol. 2020 Jan 23;3:44. doi: 10.1038/s42003-020-0768-9 (PMC6978410; doi:10.1038/s42003-020-0768-9)
Supplement: Supplementary file 1 — Supplementary Information [file 42003_2020_768_MOESM1_ESM.pdf]

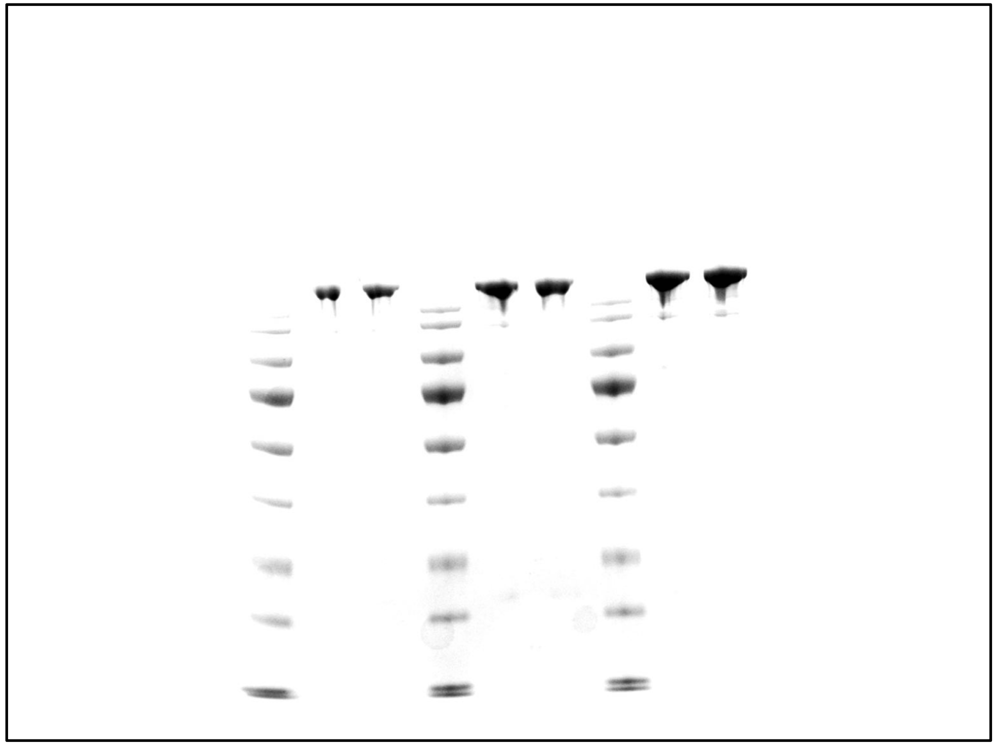

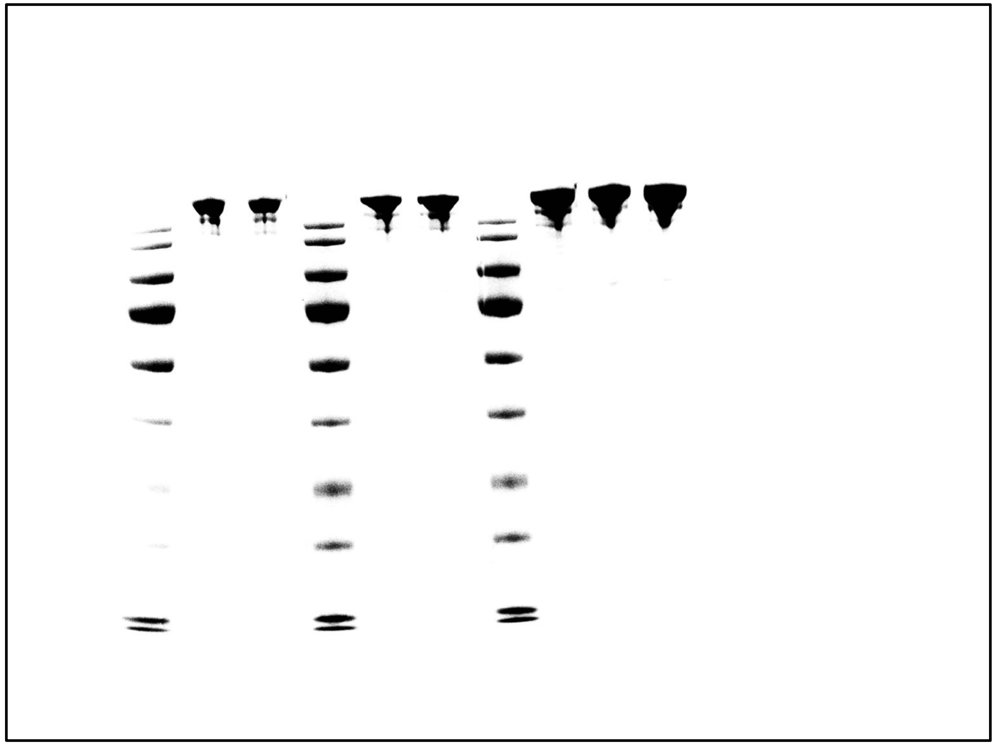

Supplementary Figure 1. Original images used to create figure 2A.

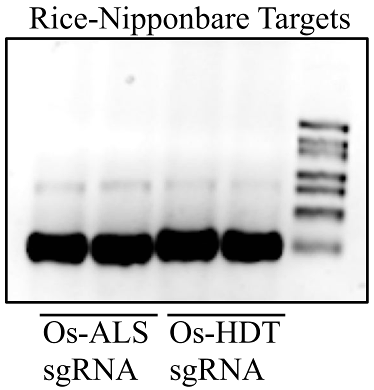

Supplementary Figure 2.
In vitro transcription of the OsALS and OsHDT sgRNAs. A PCR fragment containing the promoter sequence of T7, the sgRNA sequence, and the poly-T terminator signal were in vitro transcribed using the in vitro transcription kit. The sgRNA was separated on a 1.2% agarose gel.

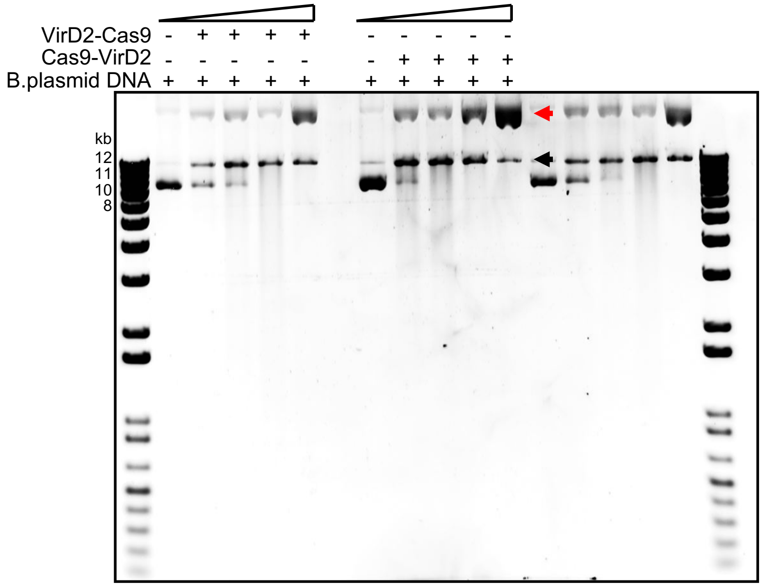

Supplementary Figure 3. Original image used to create figure 2B.

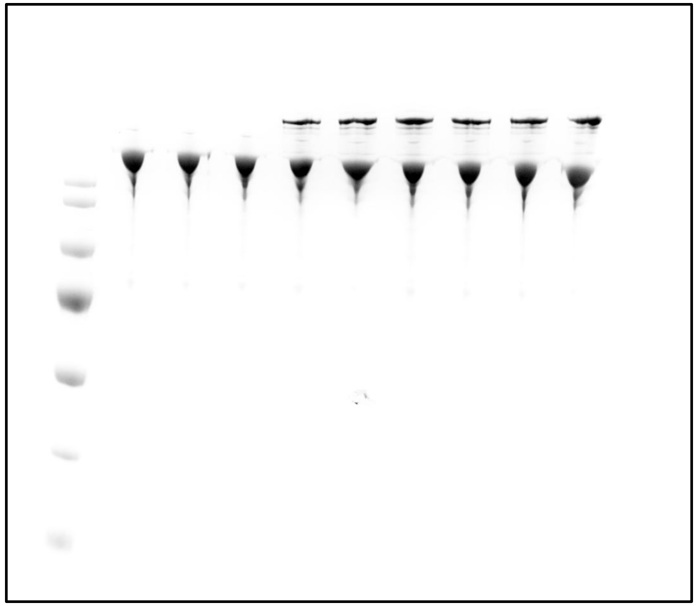

Supplementary Figure 4. Original image used to create figure 2C.

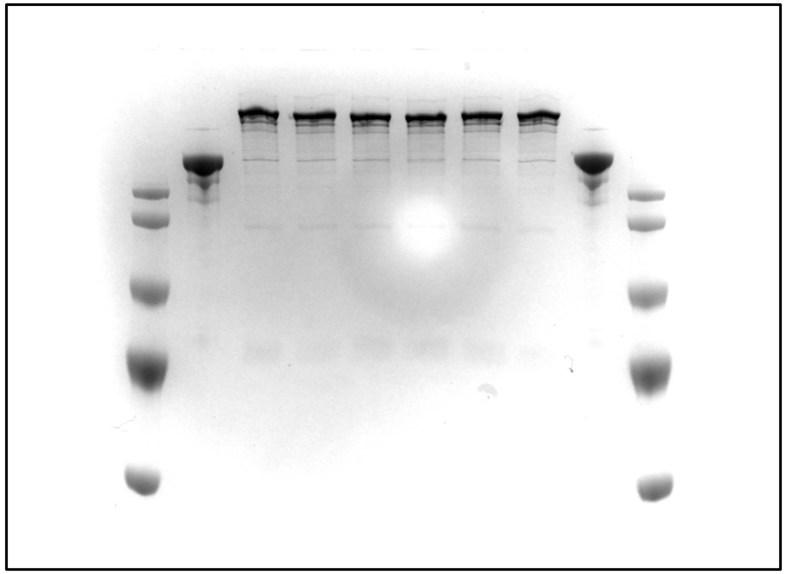

Supplementary Figure 5. Original image used to create figure 2D.

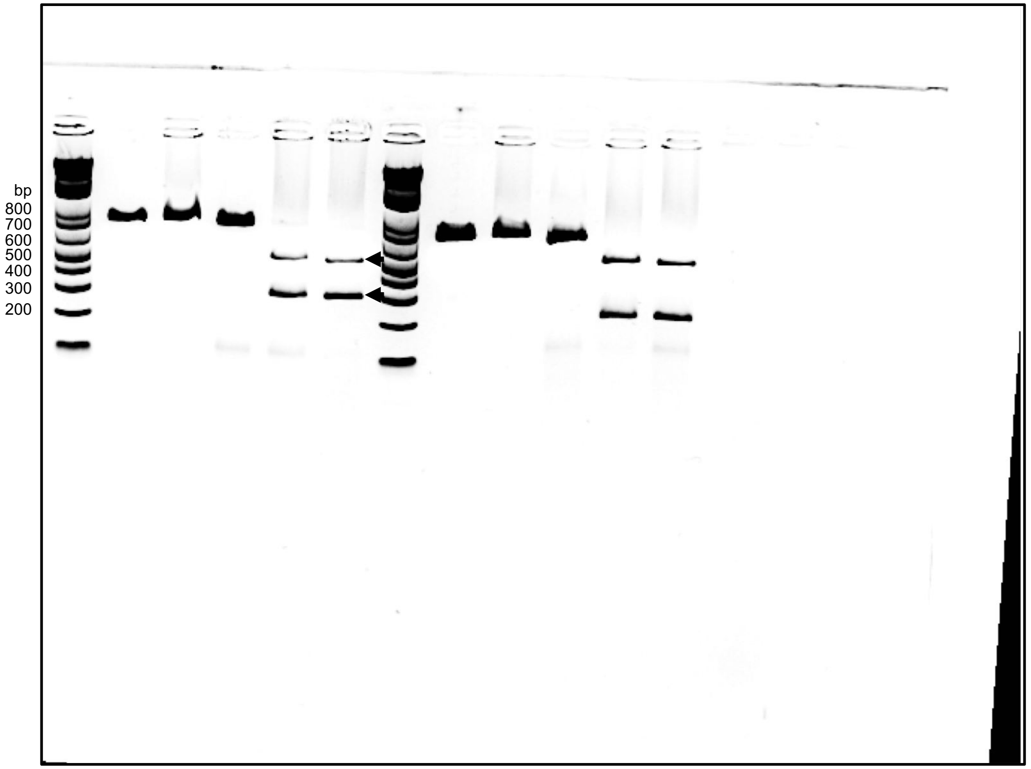

Supplementary Figure 6. Original image used to create figure 2E and 2F.

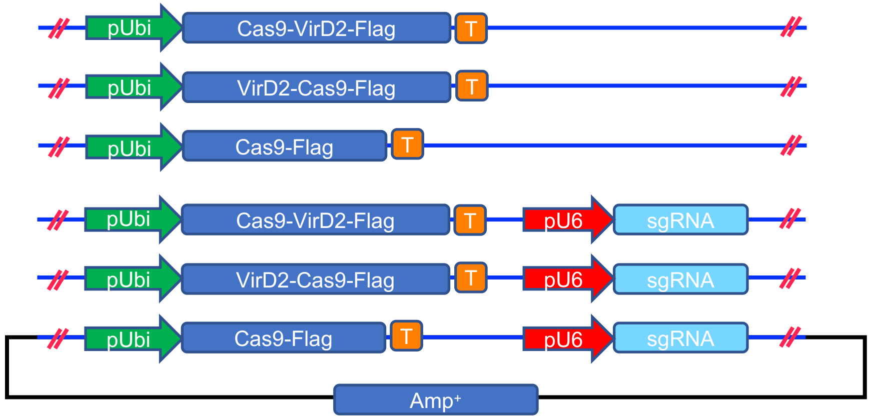

Supplementary Figure 7.
Schematic diagram of the rice protoplast-compatible plasmids. The plant codon-optimized Cas9-VirD2 and VirD2-Cas9 complexes were cloned into protoplast-compatible plasmids under the control of the UBIQUITIN promoter followed by a terminator, and sgRNAs were cloned under the U6 promoter followed by a termination signal.

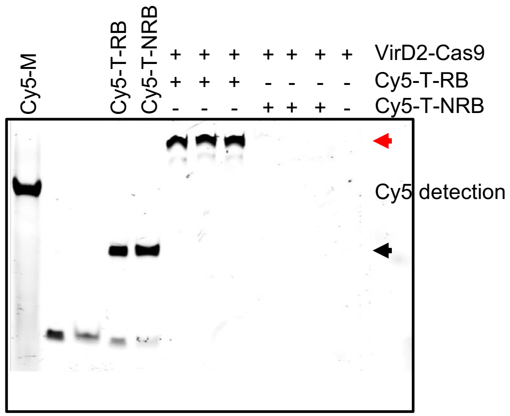

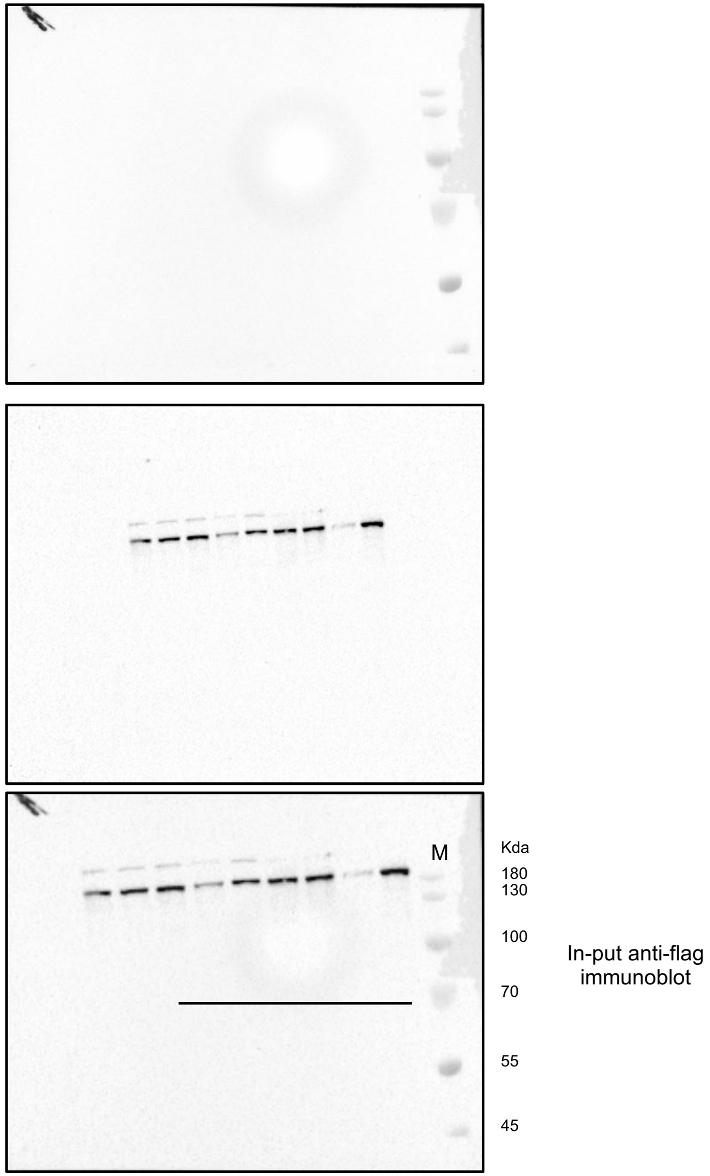

Supplementary Figure 8. Original images used to create figure 3A, the immunoblot was merged with membrane image.

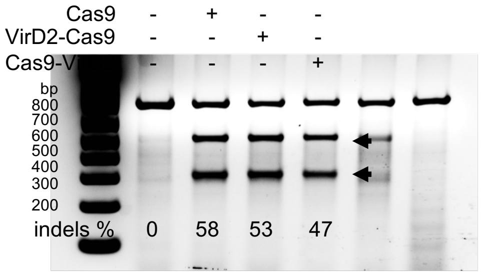

Supplementary Figure 9. Original image used to create figure 3C.

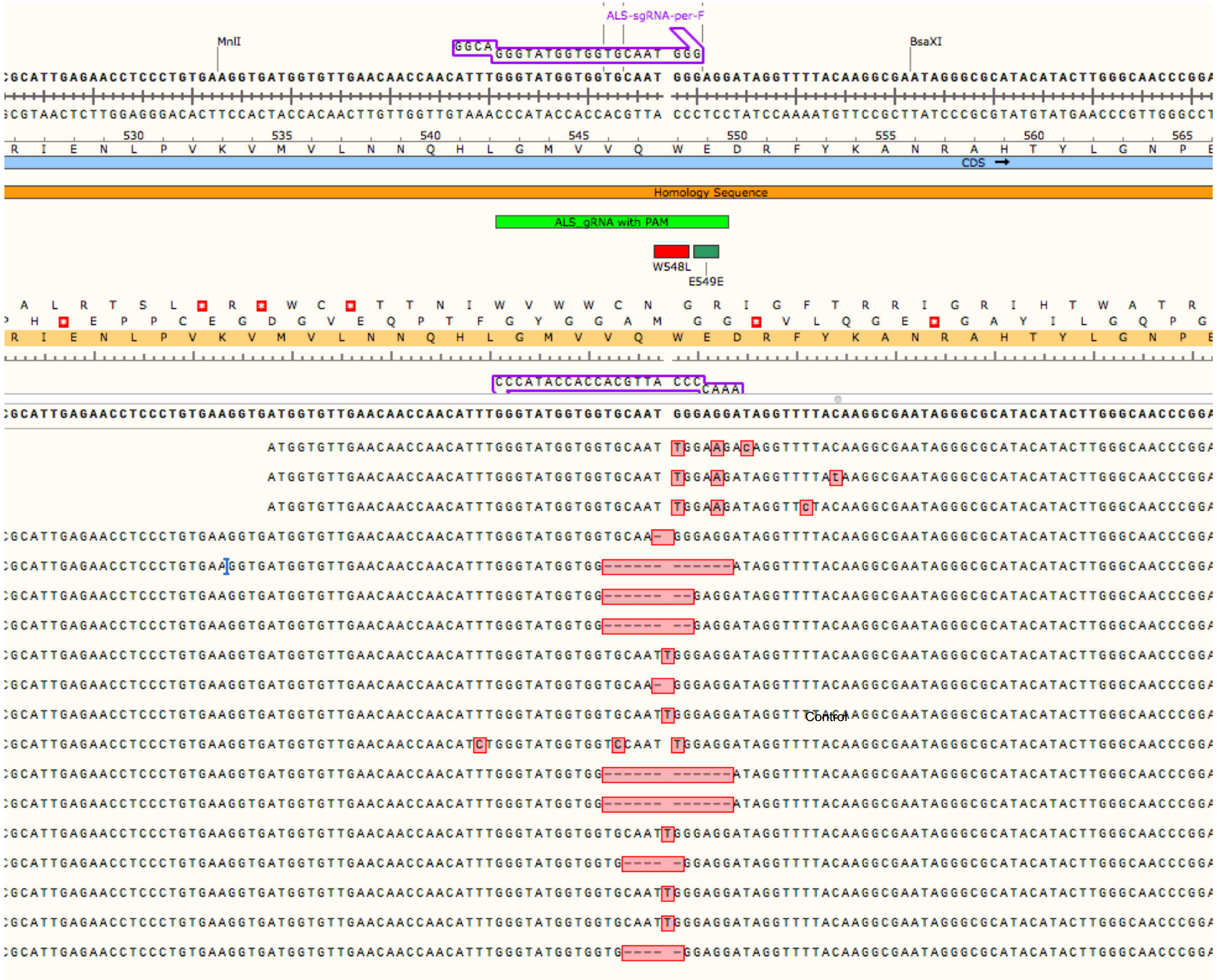

Supplementary Figure 10. Original alignment used to create figure D.

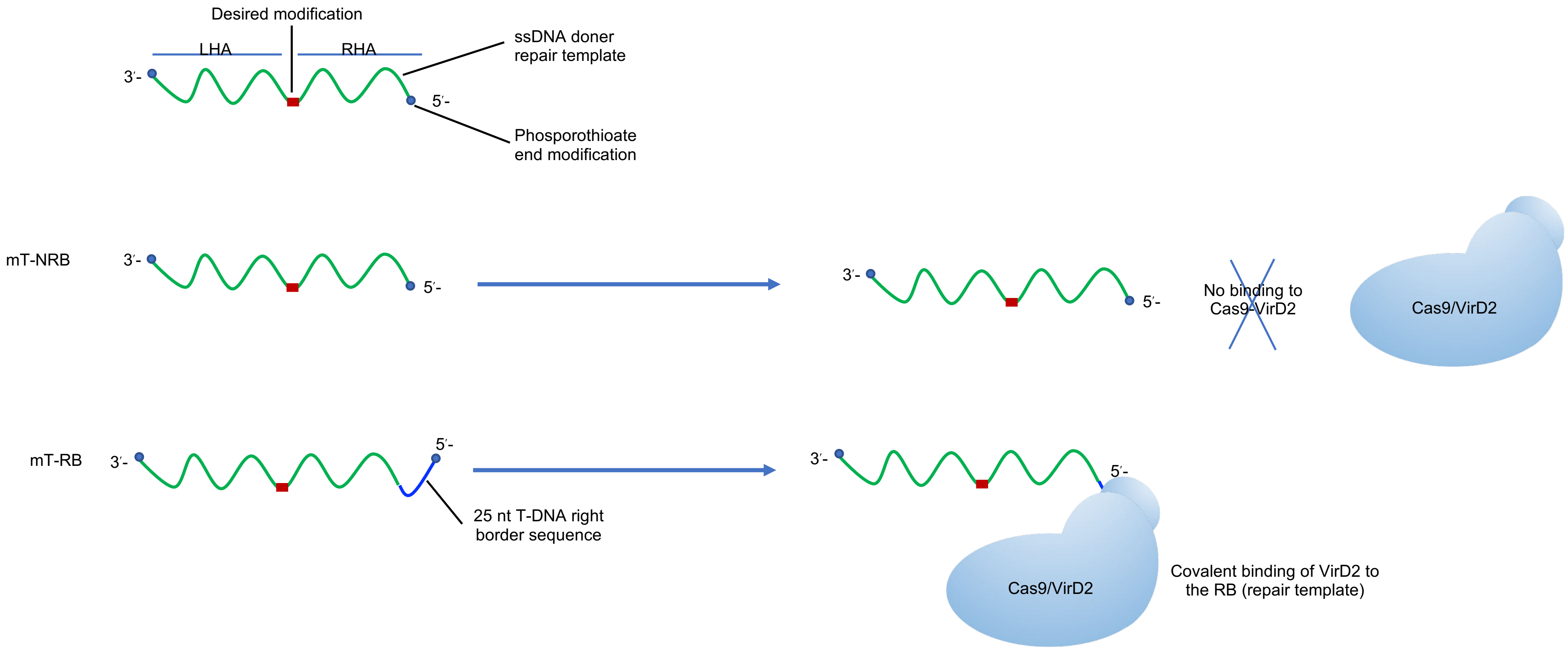

Supplementary Figure 11.
Schematic of the repair template design. The desired modification (red) is flanked by right and left homology arms. The end of the ssDNA repair templates is modified with phosphorothioate (blue circles). The RB sequence is represented by a blue line. The repair template with the RB sequence can covalently bind to Cas9-VirD2 or VirD2-Cas9.

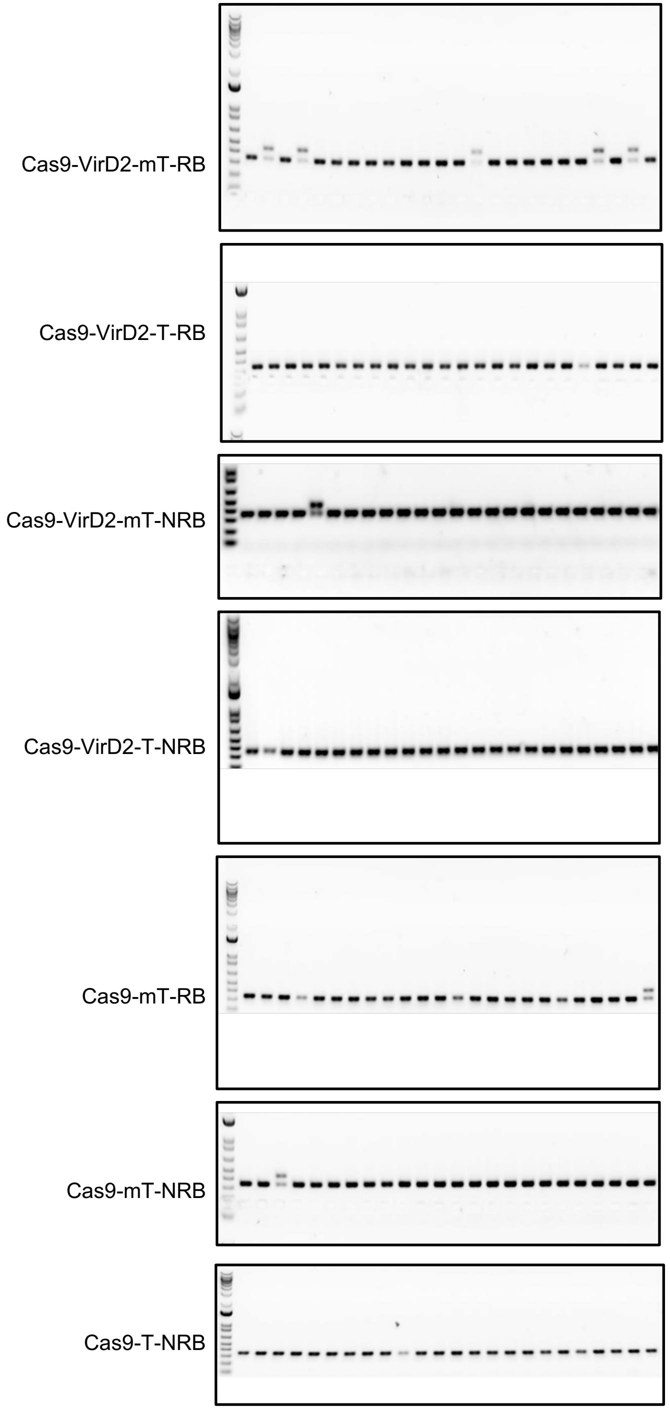

Supplementary Figure 12. Original images used to create figure 4B.

A

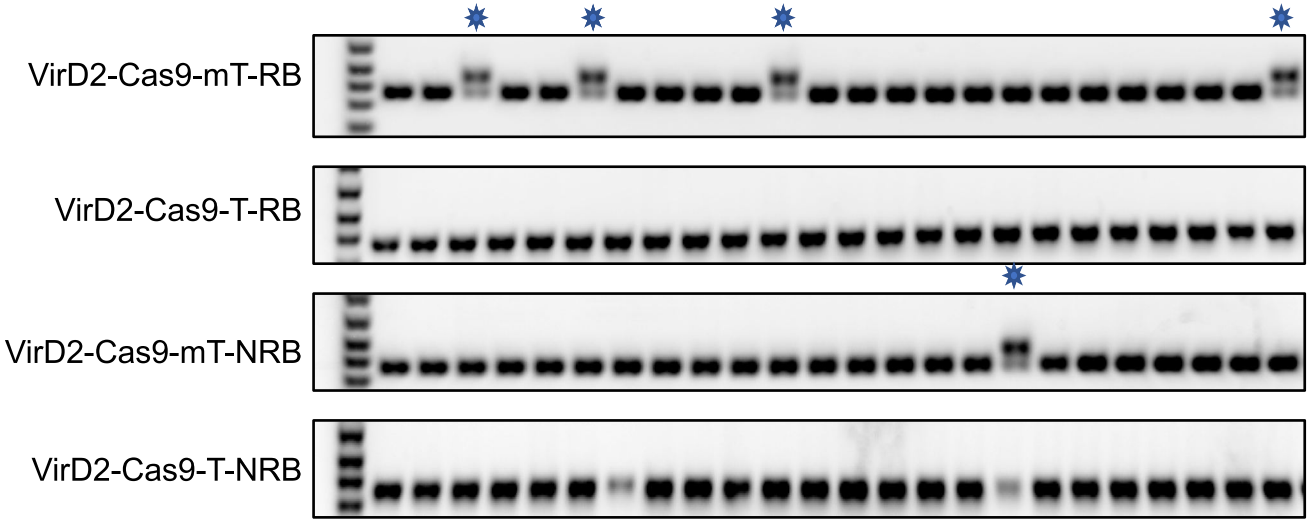

B

Clones and repair templates	Repaired +ive calli	Total no of calli	Calli with modified allele %
VirD2-Cas9 + mT-RB	5	24	16.6%
VirD2-Cas9+ T-RB	0	24	0%
VirD2-Cas9+ mT-NRB	1	24	4.1%
VirD2-Cas9+ T-NRB	0	24	0%

Supplementary Figure 13.
Allele-specific PCR confirmation of the exact repair in rice callus. (a) The allele-specific PCR from the DNA extracted from calli bombarded with phosphorothioate-modified repair template and unmodified repair templates (mT-RB, T-RB, mT-NRB, T-NRB) and VirD2-Cas9. (b) representation of the enhanced rate of HDR with phosphorothioate-modified repair template with RB (mT-RB) with VirD2-Cas9. The phosphorothioate-modified repair template with RB (mTRB) in combination with VirD2-Cas9, enhanced the rate of HDR.

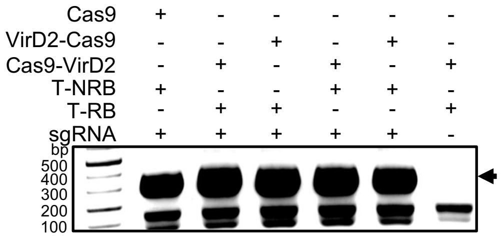

Supplementary Figure 14.
Allele-specific confirmation of the exact repair in the rice callus. The allele-specific PCR from the DNA extracted from a pool of 100 selected calli bombarded with phosphorothioate-modified repair templates and unmodified repair templates with and without RB sequence (T-NRB, mTNRB, T-RB and mT-RB) with Cas9 or VirD2-Cas9 or Cas9-VirD.

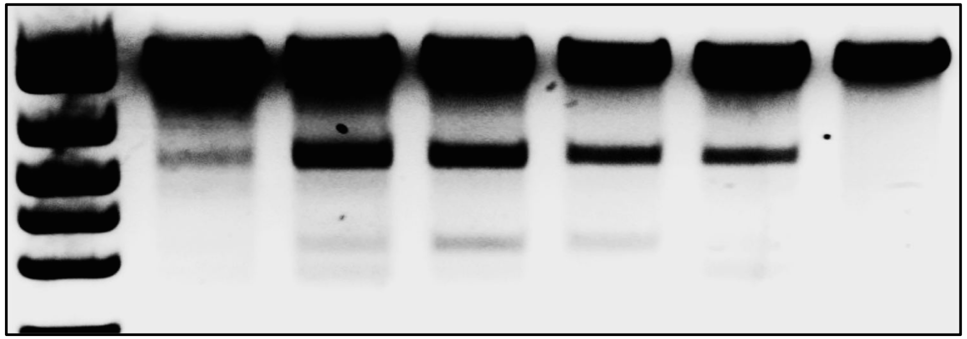

Supplementary Figure 15. Original images used to create figure 4C.

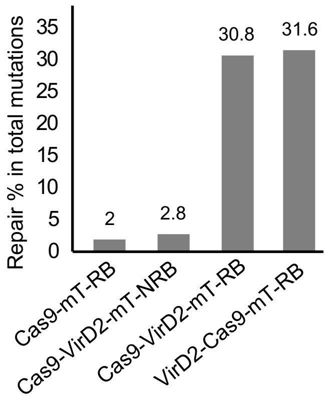

Supplementary Figure 16.
Comparison of the repair rate in the genomic DNA of the calli transformed with Cas9, Cas9-VirD2, and VirD2-Cas9. Target-flanking PCR product from the DNA of pooled (N = 100 calli) was subjected to amplicon sequencing using the TruSeq platform. Amplicon sequencing of the target-flanking PCR product confirmed a 15 fold increase of the desired edits at the target locus in the calli bombarded with the repair templates with the RB and VirD2-Cas9 or Cas9-VirD2 compared to a 2- and 2.8-fold repair rate with Cas9 and VirD2-Cas9 with a repair template lacking the RB, respectively.

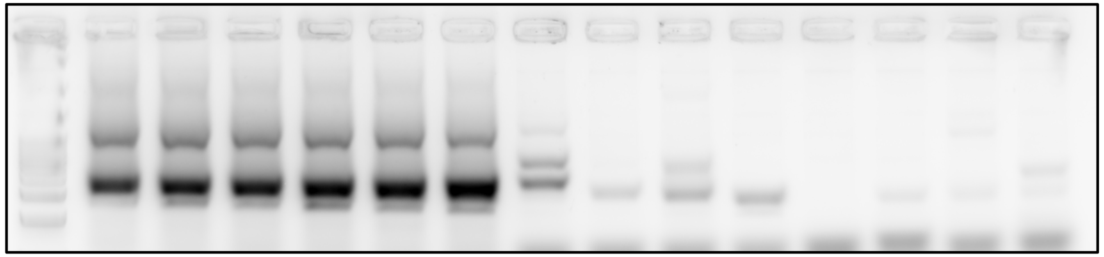

Supplementary Figure 17. Original image used to create figure 5B.

57692.1	69099.05	26987.55	11435.77	2356.87	4645.33
66853.71	74946.35	29532.66	17612.59	1113.113	3340.205
67787.64	69473.64	9648.569	10693.45	3604.426	4736.962
64111.15	71173.01	22056.26	13247.27	2358.136	4240.832
4554.938	2672.528	8834.874	3101.589	1017.075	637.9375

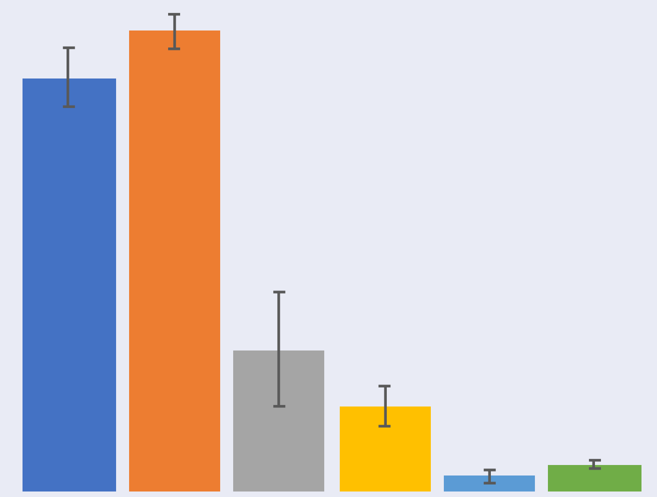

Supplementary Figure 18. Data used used to create figure 5C.

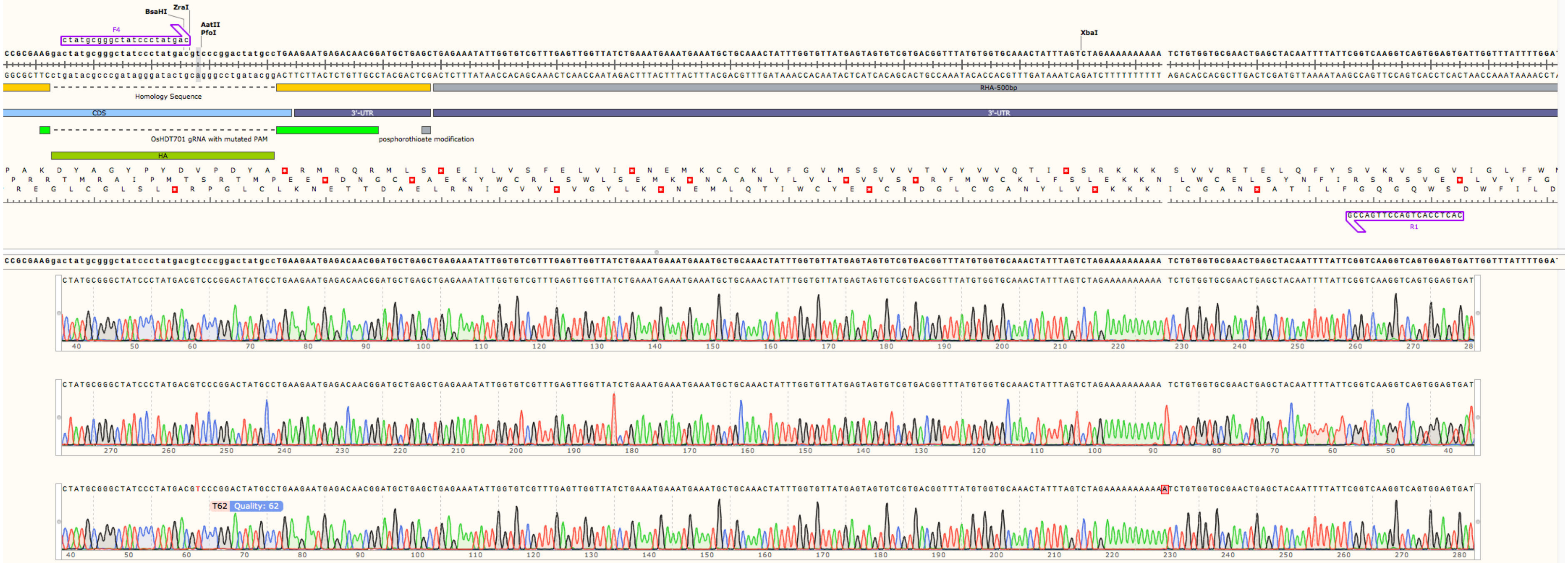

Supplementary Figure 19. Original alignment used to create figure 5D.

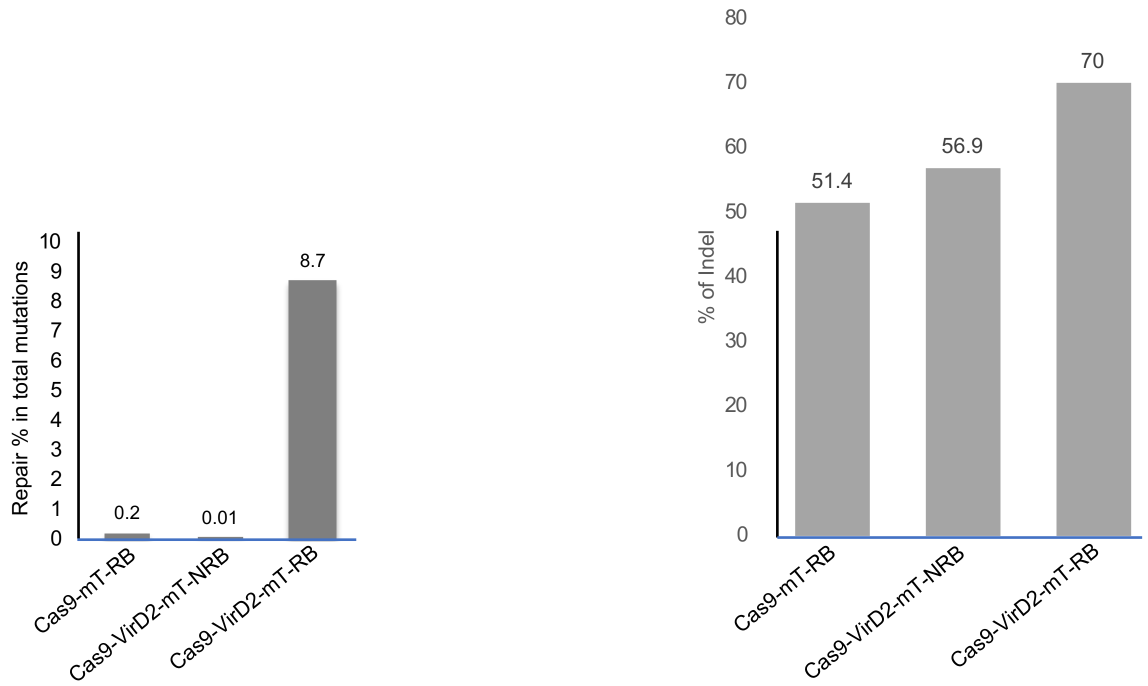

Supplementary Figure 20.
(a) Comparison of the HA epitope insertion rate in the genomic DNA of the calli transformed with Cas9, Cas9-VirD2, and VirD2-Cas9. Target-flanking PCR product from the DNA of pooled calli (N, 100) was subjected to amplicon sequencing using the TruSeq platform. Amplicon sequencing of the target flanking PCR product confirmed an 8-fold increase of the HA epitope insertion at the target locus in the calli bombarded with the repair templates with the RB and VirD2-Cas9 compared to a 0.2- and 0.1-fold repair rate with Cas9 and VirD2-Cas9 with repair templates lacking the RB, respectively. (b) The % indels is shown to confirm the normal targeting by Cas9-VirD2 and VirD2-Cas9 compared to Cas9.

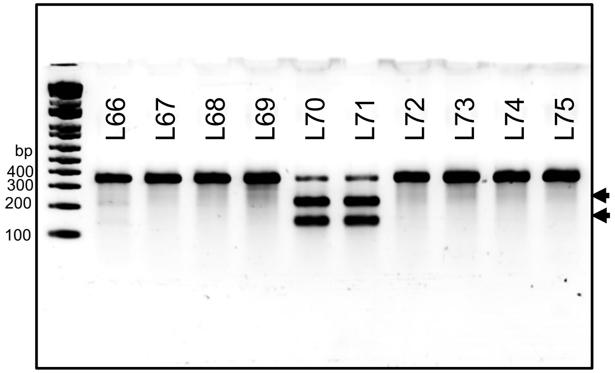

Supplementary Figure 21. Original image used to create figure 6A.

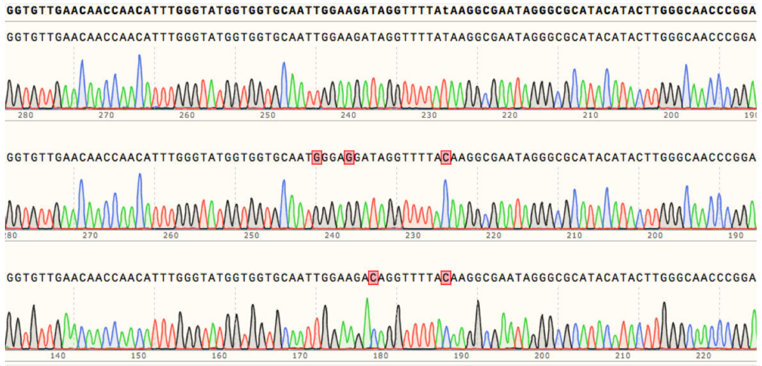

Supplementary Figure 22. Original alignment used to create figure 6B and C.

A

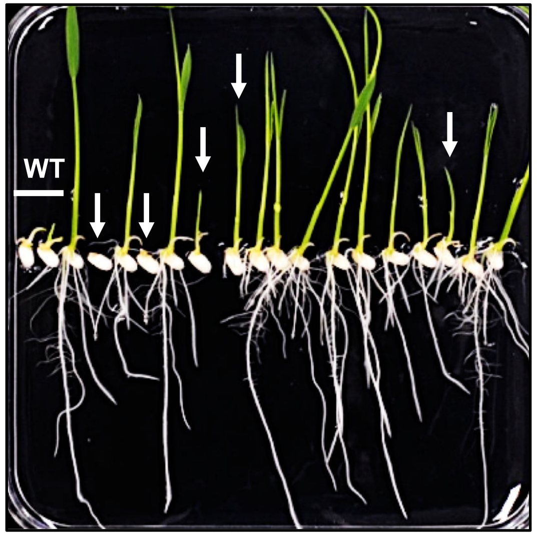

70 - progeny plants - BS media

B

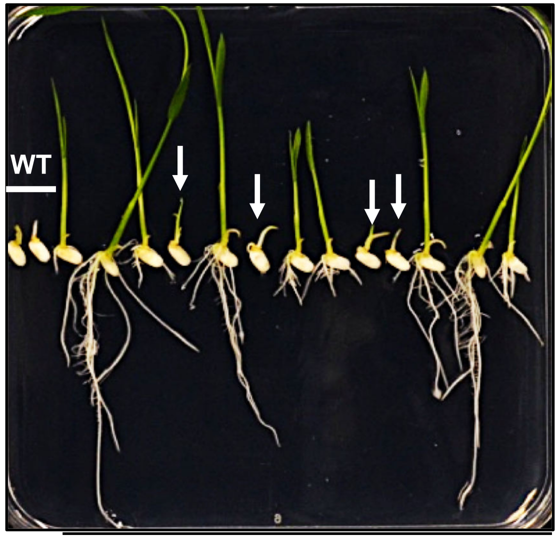

121 - progeny plants - BS media

C

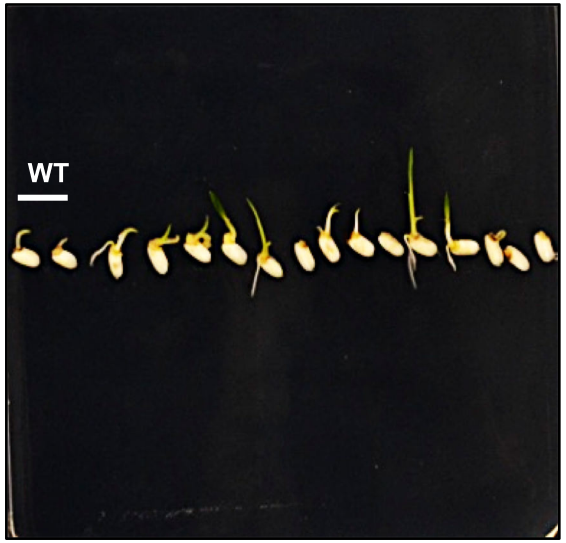

CNT - BS media

D

Plants progeny	Total no of seedling	BS-resistance seedlings	BS-sensitive seedling	BS-resistance frequency
Line 70	17	12	5	70.5%
Line 121	13	9	4	69.23%
CNT	14	0	0	0

Supplementary Figure 23.
Confirmation of the herbicide resistance and heritability of the modified allele in the T1 progenies of rice plants. (a, b, and c) Seeds collected from line 70 and line 121 were germinated on vertical plates with ó MS media containing bispyribac. Seeds from the plants expressing only Cas9 were used as a control. Plants with the modified ALS allele showed proper root and shoot development and heritability of the herbicide resistance. Plants expressing only Cas9 with repair templates (CNT) were used as control. In a, b, and c two seeds from WT plants were used as control. (d) Table of the herbicide resistance allele heritability.

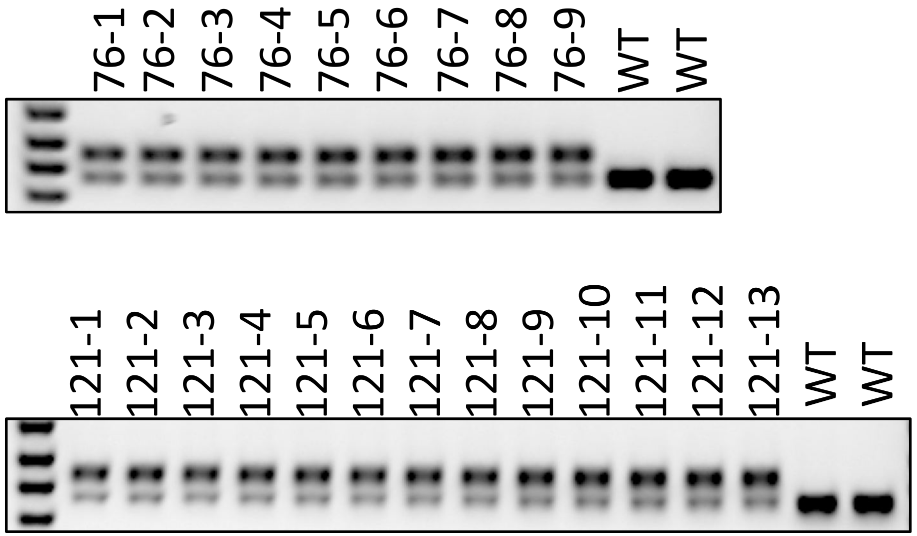

Supplementary Figure 24.
Allele-specific PCR confirmation of the herbicide resistance allele in the T1 progeny of line 76 and line 121. DNA was extracted from the germinated plants and subjected to modified ALS allele-specific PCR. The amplification of the exact size 417bp fragment with the allele specific PCR (indicated by the arrow head) confirmed the presence of the herbicide resistance allele. The lower band is a nonspecific PCR amplification and present in all samples.

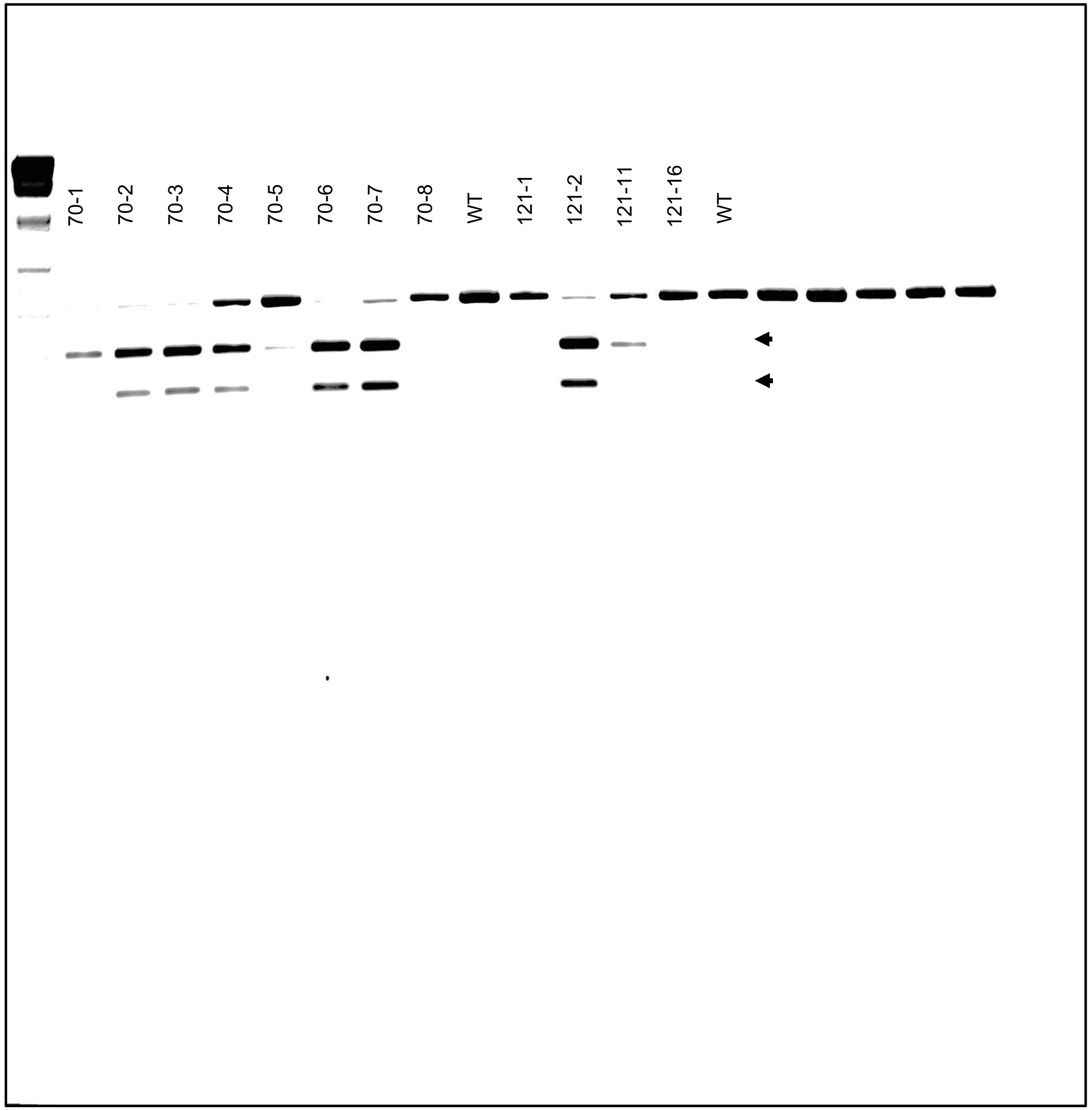

Supplementary Figure 25. Original image used to create figure 7b.

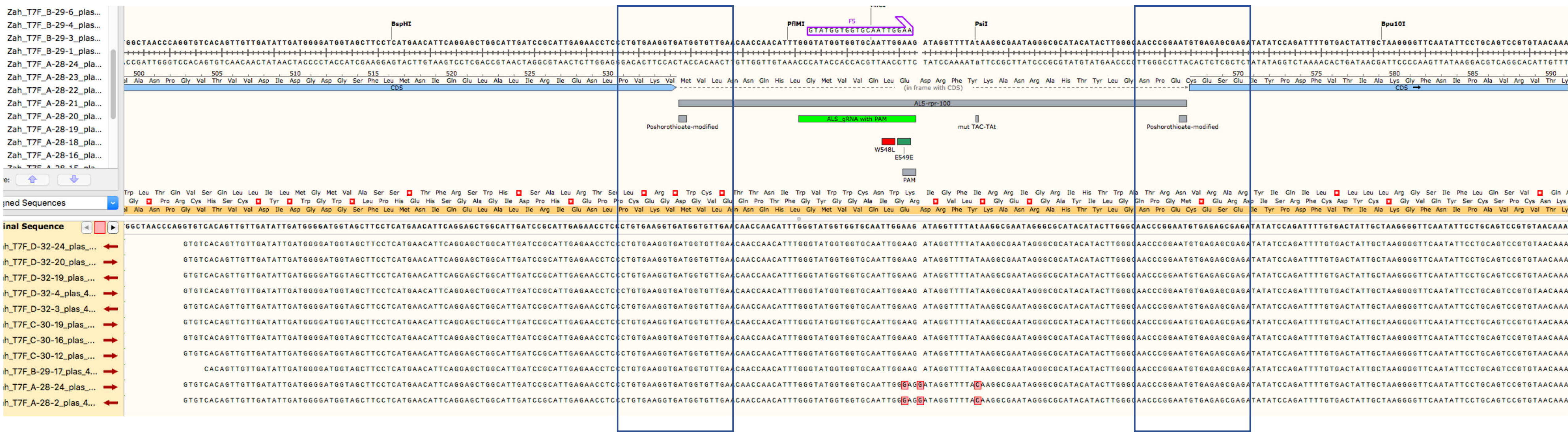

Supplementary Figure 26. Original image used to create figure 7C and D. The precise nucleotide changes are shown, and the Junctions (boxes) confirmed the exact insertion with out any extra nucleotide changes at the repaired junction sites.

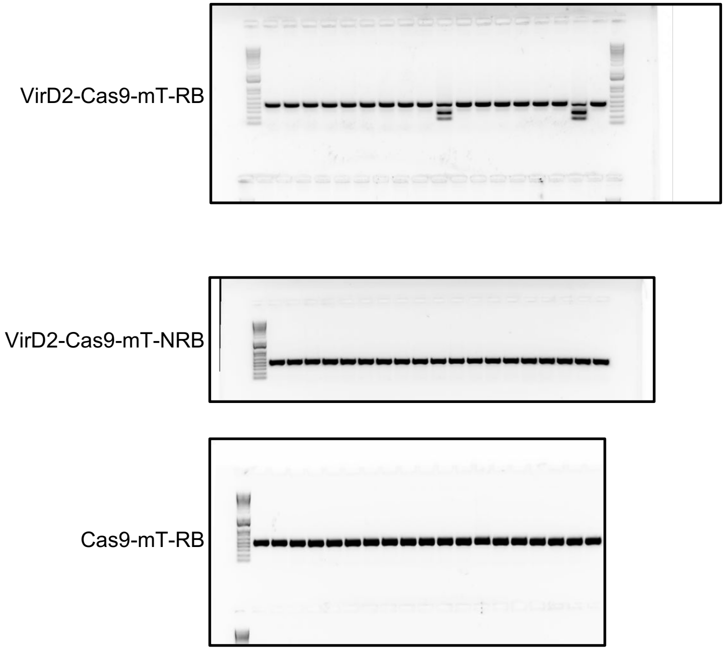

Supplementary Figure 27. Original images used to create figure 8B.

EcoRI confirmation part of the ccd7 figure

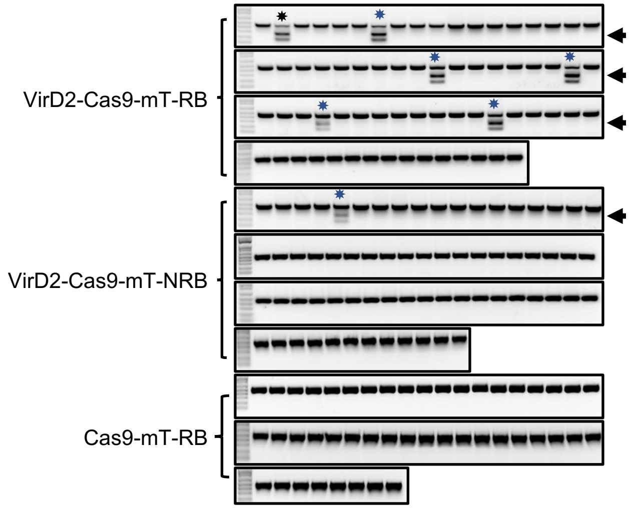

Supplementary Figure 28.
EcoRI digestion to evaluate targeted modification of CCD7. Target-flanking 445 bp PCR product digested with EcoRI produced 270 + 175 bp fragments (indicated by arrowheads) in the successfully edited plants regenerated from calli bombarded with repair templates with mT-RB and Cas9-VirD2 or VirD2-Cas9. Repair templates without mT-NRB with VirD2-Cas9 and Cas9 with mT-RB repair templates were used as control. Lines confirmed by EcoRI digestion are indicated with asterisks.

```
     GTGGCAAAACTAGAGGTGCCAAAGAACCTCACTTTTCCAATGGGATTCCATGGTTTCTGGGGAGATGAATGAGCAT WT

1567 GTGGCAAAACTAGAGGTGCCAAAGAACCTCACTTTCCTAATGGAATTCCCTGGTTTCTGGGGAGATGAATGAGCAT Repaired allele
     GTGGCAAAACTAGAGGTGCCAAAGAACCTCACTT---CAATGGGATTCCATGGTTTCTGGGGAGATGAATGAGCAT Mutated allele


     GTGGCAAAACTAGAGGTGCCAAAGAACCTCACTTTTCCAATGGGATTCCATGGTTTCTGGGGAGATGAATGAGCAT WT

1588 GTGGCAAAACTAGAGGTGCCAAAGAACCTCACTTTCCTAATGGAATTCCCTGGTTTCTGGGGAGATGAATGAGCAT Repaired allele
     GTGGCAAAACTAGAGGTGCCAAAGAACCTCACTTTTC-AATGGGATTCCATGGTTTCTGGGGAGATGAATGAGCAT Mutated allele


     GTGGCAAAACTAGAGGTGCCAAAGAACCTCACTTTTCC-AATGGGATTCCATGGTTTCTGGGGAGATGAATGAGCAT WT

1606 GTGGCAAAACTAGAGGTGCCAAAGAACCTCACTTTCCT-AATGGAATTCCCTGGTTTCTGGGGAGATGAATGAGCAT Repaired allele
     GTGGCAAAACTAGAGGTGCCAAAGAACCTCACTTTTCCCAATGGGATTCCATGGTTTCTGGGGAGATGAATGAGCAT Mutated allele
```

Supplementary Figure 29.
Sanger sequence alignment of the T0 plants. DNA was extracted from three independent T0 plants. The target sequencing results confirm the bi-allelic genotype, including one precisely edited allele in these three plants.

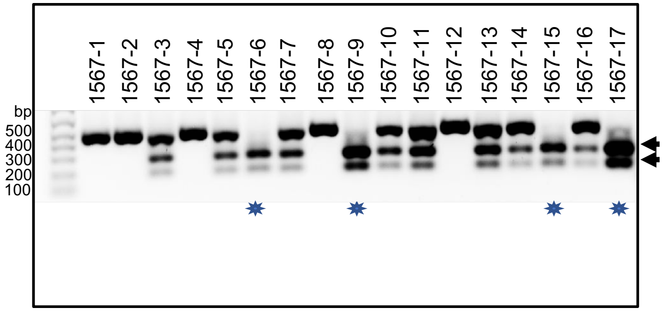

Supplementary Figure 30. Original image used to create figure 8D.

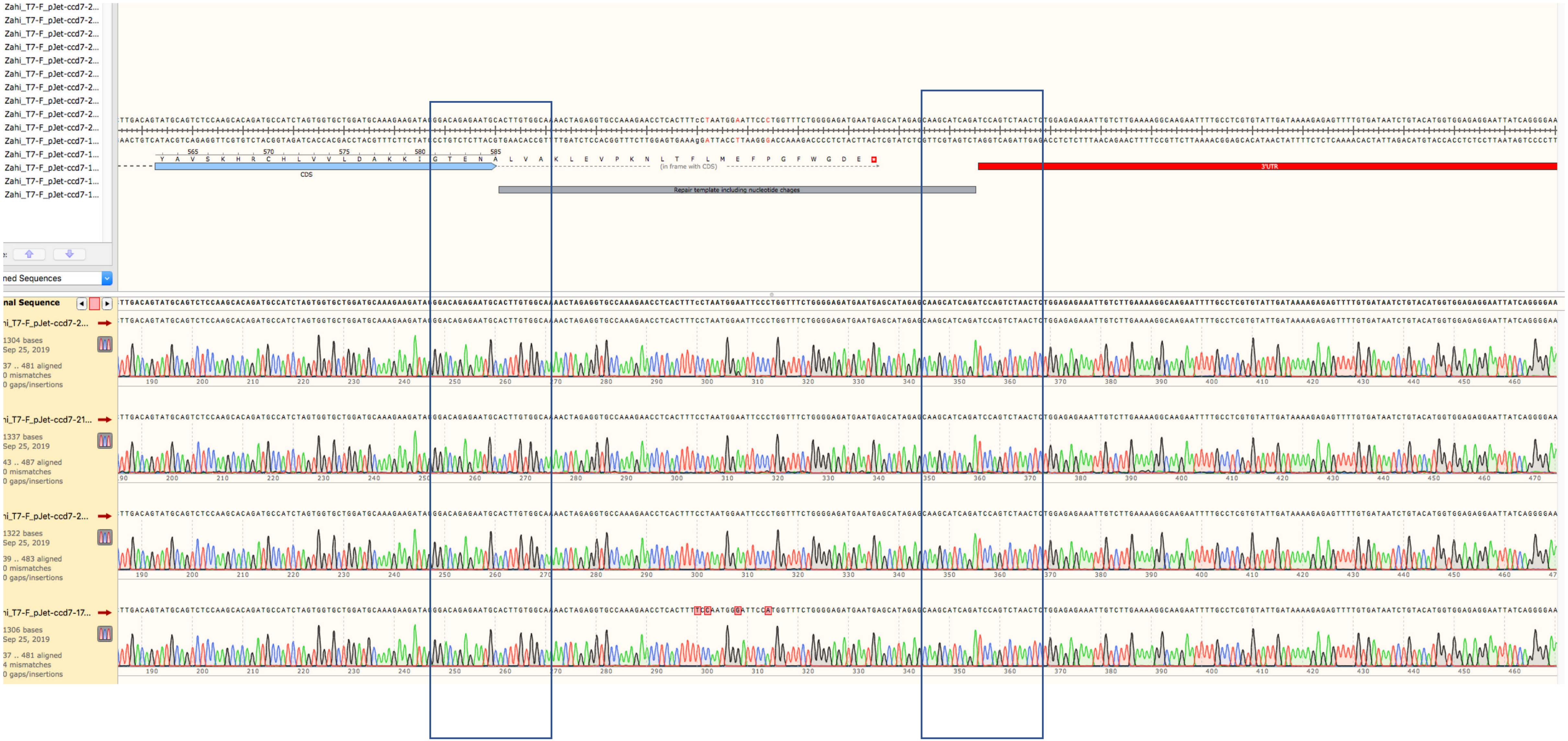

Supplementary Figure 31. Original image used to create figure 8E. The precise nucleotide changes are shown, and the Junctions (boxes) confirmed the exact insertion with out any extra nucleotide changes at the repaired junction sites.

Supplementary Table 1.

Clones and repair templates	Repaired +ive plants	Total T0 plants	Repair frequency
VirD2-Cas9+ mT-RB	6	69	8.6%
VirD2-Cas9+ mT-NRB	1	64	1.5%
Cas9 + mT-RB	0	46	0%

Supplementary Table 1. Comparison of the VirD2-Cas9 with T-RB, VirD2-Cas9 with T-NRB and Cas9 with mT-RB for gene targeting.

Supplementary Table 2.

Total T1 seedlings germinated	17
Total EcoRI digested	11
Homozygous for EcoRI digestion	4
Heterozygous for EcoRI digstion	8
Total EcoRI un-digested	5

Supplementary Table 2. Representing the heritability of the EcoRI site targeted insertion.

Supplementary Table 3. Primers used in this study**.**

primers name	sequence (5ˊ ---- 3ˊ)	Usage
VirD2_F<br>VirD2_R	CACC ATGCCCGATCGAGCTCAAGTTATCATTCGC<br>ggg CTATCTCCTATTTCCCCCACGCCCGTCCTTG	to amplify form agrobacterium GV3101
pt28VecV2C-F<br>pt28VecV2C-R	Atggacaagaagtacagcatcggcctggac<br>gcggccgcaagcttgtcgacggagctcgaa	to amplify Vector and Cas9 for making pet28a-VirD2-Cas9-for protein expression in Ecoli by Gibson assembly
pt28V2N-F<br>pt28V2N-R	gtcgacaagcttgcggccgcATGCCCGATCGAGCTCAAG<br>atgctgtacttcttgtccatACTTCCACCTCCCCCACTTC	to amplify VirD2-linker for making pet28a-VirD2-Cas9-for protein expression in Ecoli by Gibson assembly
pt28Vec-CV2-F<br>pt28Vec-CV2-R	taagcggccgcactcgagcaccaccaccac<br>acaggcgtagtcgggcacgtcgtaggggta	to amplify Vector and Cas9 for making pet28a-Cas9-VirD2 for protein expression in Ecoli by Gibson assembly
pt28V2N-F<br>pt28V2N-R	gtcgacaagcttgcggccgcATGCCCGATCGAGCTCAAG<br>atgctgtacttcttgtccatACTTCCACCTCCCCCACTTC	to amplify VirD2-linker for making pet28a-VirD2-Cas9-for protein expression in Ecoli by Gibson assembly
HDT-HA-100-RPR-F mT-NRB	CTCACTCGAAGGCCAAGCACCCCGCGAAGgactatgcgggctatccctatgacgtcccggactatgccTGAAGAATGAGACAACGGATGCTGAGC	HA epitope insertion repair template at HDT site
HDT-HA-100-RPR-R mT-NRB	GCTCAGCATCCGTTGTCTCATTCTTCAggcatagtccgggacgtcatagggatagcccgcatagtcCTTCGCGGGGTGCTTGGCCTTCGAGTGAG	HA epitope insertion repair template at HDT site
RB-HDT-HA-100-AS-RPR-F mT-RB	CTCACTCGAAGGCCAAGCACCCCGCGAAGgactatgcgggctatccctatgacgtcccggactatgccTGAAGAATGAGACAACGGATGCTGAGCtgacaggatatattggcgggtaaacatgc	HA epitope insertion repair template at HDT site
RB-HDT-HA-100-S-RPR-R mT-RB	gcatgtttacccgccaatatatcctgtcaGCTCAGCATCCGTTGTCTCATTCTTCAggcatagtccgggacgtcatagggatagcccgcatagtcCTTCGCGGGGTGCTTGGCCTTCGAGTGAG	HA epitope insertion repair template at HDT site
ALS-rpr-100 mT-NRB	ATGGTGTTGAACAACCAACATTTGGGTATGGTGGTGCAATTGGAAGATAGGTTcTAtAAGGCGAATAGGGCGCATACATACTTGGGCAACCCGGAAT	Herbicide allele repair template at ALS site
RB-ALS-rpr-100-AS mT-RB	gtttacccgccaatatatcctgtcaTTCCGGGTTGCCCAAGTATGTATGCGCCCTATTCGCCTTaTAgAACCTATCTTCCAATTGCACCACCATACCCAAATGTTGGTTGTTCAACACC	Herbicide allele repair template at ALS site
RB-ALS-rpr-100-S mT-NRB	gtttacccgccaatatatcctgtcaGGTGTTGAACAACCAACATTTGGGTATGGTGGTGCAATTGGAAGATAGGTTcTAtAAGGCGAATAGGGCGCATACATACTTGGGCAACCCGGAA	Herbicide allele repair template at ALS site
Cas9V-pRG-Cas9-F	agcagccgacaagaagtacagcatcggcctggacatcggc	Cas9-VirD2 in to plant vector by Gibson cloning
Cas9V-pRG-Vec-R2	ttcttgtcggctgctgggactccgtggataccgaccttc	Cas9-VirD2 in to plant vector by Gibson cloning
CasV-pRG-Cas9-Vec-R1	TCAGGCATgtcgcctcccagctgagacaggtcgatccgt	Cas9-VirD2 in to plant vector by Gibson cloning
CasV-pRG-V2-F	aggcgacATGCCTGACAGAGCACAGGTTATAATTCGTATAG	Cas9-VirD2 in to plant vector by Gibson cloning
CasV-pRG-V2-R	cggccttttTCTTCTGTTCCCTCCTCTTCCATCTTTGGATCTTTC	Cas9-VirD2 in to plant vector by Gibson cloning
CasV-pRG-Vec-F	ACAGAAGAaaaaggccggcggccacgaaaaaggccggccag	Cas9-VirD2 in to plant vector by Gibson cloning
VCas9-pRGVecF1-Cas9F	GAACAGAAGAgacaagaagtacagcatcggcctggacatcggcacc	Cas9- VirD2 in to plant vector by Gibson cloning

VCas9-pRG-Cas9-R	ggccttttgtcgcctcccagctgagacaggtcgatccgt	Cas9- VirD2 in to plant vector by Gibson cloning
VCas9-pRG-V2-R	acttcttgtcTCTTCTGTTCCCTCCTCTTCCATCTTTggatctttc	Cas9- VirD2 in to plant vector by Gibson cloning
VCas9-pRG-V2F	agcagccATGCCTGACAGAGCACAGGTTATAATTCGTATAG	Cas9- VirD2 in to plant vector by Gibson cloning
VCas9-pRG-Vec-F2	aggcgacaaaaggccggcggccacgaaaaaggccggccag	Cas9- VirD2 in to plant vector by Gibson cloning
VCas9-pRG-Vec-R	TCAGGCATggctgctgggactccgtGGATACCGACCTTCC	Cas9- VirD2 in to plant vector by Gibson cloning
ALS-F4	GTGTCACAGTTGTTGATATTG	Oligo sequence for T7EI assay and amplicon sequencing
ALS-R4	GGACGATGATATCCAACAAG	Oligo sequence for T7EI assay and amplicon sequencing
ALS-F5	GTATGGTGGTGCAATTGGAA	Herbicide resistance allele specific F primer
ALS-R10	CTTAGTTATTGATAGAGC	R primer for allele specific PCR
HDT-F5	GGAGTATTACGTAAGGAAATGATTC	Oligo sequence for T7EI assay and amplicon sequencing
HDT-R5	CTAGACTAAATAGTTTGCACCAC	Oligo sequence for T7EI assay and amplicon sequencing
CCD7-rpr-100 mT-NRB	ACTTGTGGCAAAACTAGAGGTGCCAAAGAACCTCACTTTcCTAATGGAATTCCCTGGTTTCTGGGGAGATGAATGAGCATAGAGCAAGCATCAGA	allele repair template at CCD7 site
RB-CCD7-rpr-100-AS mT-RB	gcatgtttacccgccaatatatcctgtcaTCTGATGCTTGCTCTATGCTCATTCATCTCCCCAGAAACCAGGGAATTCCATTAGAAAAGTGAGGTTCTTTGGCACCTCTAGTTTTGCCACAAGT	allele repair template at CCD7 site
RB-CCD7-rpr-100-S mT-NRB	gcatgtttacccgccaatatatcctgtcaACTTGTGGCAAAACTAGAGGTGCCAAAGAACCTCACTTTTCTAATGGAATTCCCTGGTTTCTGGGGAGATGAATGAGCATAGAGCAAGCATCAGA	allele repair template at CCD7 site
CCD7-RT-F	GAGGATGGTGGCTATGTTCTTCT	Oligo sequence for EcoRI assay and Sanger sequencing
CCD7-RT-R	AGTAGTTATTTGGTTCCCCTGAT	Oligo sequence for EcoRI assay and Sanger sequencing
T-RB-60b	ctactgtgaAAACTATCAGTGTTTGACAGGATATATTGGCGGGTAAACCTAAGAGAAttg	Binding assay
T-NRB-60b	ctactgtgaAAACTATCAGTGTTatgctatgaATATTGGCGGGTAAACCTAAGAGAAttg	Binding assay Negative control
Cy5-T-RB	gcatgtttacccgccaatatatcctgtcaACTTGTGGCAAAACTAGAGGTGCCAAAGAACCTCACTTTTCTAATGGAATTCCCTGGTTTCTGGGGAGATGAATGAGCATAGAGCAAGCATCAGA-Cy5	Immunoprecipitation assay
T-RB	gcatgtttacccgccaatatatcctgtcaACTTGTGGCAAAACTAGAGGTGCCAAAGAACCTCACTTTTCTAATGGAATTCCCTGGTTTCTGGGGAGATGAATGAGCATAGAGCAAGCATCAGA	Immunoprecipitation assay Negative control
Cy5-T-NRB	ACTTGTGGCAAAACTAGAGGTGCCAAAGAACCTCACTTTTCTAATGGAATTCCCTGGTTTCTGGGGAGATGAATGAGCATAGAGCAAGCATCAGA-Cy5	Immunoprecipitation assay control

Supplementary discussion. Innovative solutions like the precise engineering of loci associated with important agronomic traits are needed to produce high-yielding crops with improved traits, such as disease or stress resistance, for the growing human population [1]. In contrast to the laborious and lengthy procedures of conventional breeding, precise genome engineering through HDR may prove to be an efficient way to introduce desired alleles or sequences into the plant genome. However, HDR is quite inefficient in plants, and the requirement for a stable repair template and a DSB at a particular site in somatic cells make it more complicated [2]. We have established an efficient HDR system by developing a bimodular chimeric fusion of the Cas9 endonuclease and the Agrobacterium VirD2 relaxase, where Cas9 generates DSBs and VirD2 binds the repair template and makes it available and in close proximity to the DSB for the HDR repair machinery.

Our system would be feasible for any type of plant tissue, somatic or germline. Also, this system can be used for DNA or ribonucleoprotein (RNP) format through biolistic delivery systems or in transient assays through PEG-mediated transformation. Different strategies were shown to enhance the rate of HDR in plants but with serious limitations. For example, viral DNA replicons were developed to express the CRISPR/Cas9 machinery for producing DSBs and to provide sufficient repair DNA template. The viral replicons increased the HDR-based knock-in frequency 3- to 5-fold in rice [3]. The viral replicon-based HDR methods are a good choice in terms of repair but possibly not for plant biotechnology due to the ambiguous fate of the viral DNA replicons after HDR. These replicons might integrate in the plant genome and the virus replication in the target cell may compromise the regenerability of the edited cells. As an alternative strategy, harnessing HDR in germline cells was conducted using sequential transformation to take advantage of the cell stage-specific promoter activity for Cas9 expression and active cellular machinery to enhance the gene targeting rate by 2- to 5-fold at different loci of the *Arabidopsis thaliana* genome [4]. However, germline-based HDR cannot be generalized to all crops as the majority of crops are not compatible with floral dip transformation, and somatic embryogenesis is required to regenerate engineered plants.

Targeted DNA sequence insertion, and gene tagging with epitopes, is challenging in eukaryotes [5, 6]. With Cas9-VirD2-coupled HDR, we efficiently inserted HA epitope at the C terminus of *HDT* in rice genome. However, the rate of HDR was lower by 2-fold (up to 8%) compared to the editing of the *ALS* locus, producing 3.7- and 5.5-fold (14–23%) higher numbers of calli. This

indicates the need for optimization of the flanking homology arms to achieve efficient HDR. However, based on the ability of VirD2 to bind to a wide range of ssDNAs, the length of the homology arm can easily be increased to enhance the rate of insertion of epitopes and the desired genetic elements at the target locus. Another possibility to explain the low repair at the callus level and no recovery of regenerated plants for HA-tagged *HDT* loci may be due to the importance of the HDT gene product for cell survival and complete plant regeneration. HDT is an essential protein for chromatin modulation through histone deacetylation [7] and any editing, including HDT-HA-tagging may not allow the proper regeneration of complete plants.

Cas9-VirD2 system has several advantages to enable functional gene analysis and engineering agronomic traits in crops. The simplicity and robustness of the Cas9-VirD2 system may enable a variety of genetic outcomes including the generation of precise genome edits, ranging from a single-base replacement to insertions of multiple kilobase-long fragments, i.e. gene staking, gene tagging, and precise insertions of specific motifs or promoters. However, despite these clear advantages, the Cas9-VirD2 system might have some limitations. First, the constitutive expression of Cas9-VirD2 and the presence of the phosphorothioate end modified repair-template might result in off-target insertions and mutations in the plant genome. Second, the current strategy is dependent on biolistic delivery and tissue culture and hence it is limited to crops amenable to biolistic delivery and tissue culture. Third, the current format requires transgenesis and may face regulatory hurdles similar to other transgenic systems. Understanding the molecular underpinnings of the Cas9.VirD2 mediated repair is required before the use of the system in targeted trait improvement applications. Overall, this new method provides an alternative system for HDR-mediated precise editing of genomes for targeted engineering in plants and possesses key advantages over existing methods including viral replicons and germline-based systems.

1. Zaidi, S.S., et al., *New plant breeding technologies for food security.* Science, 2019. **363**(6434): p. 1390-1391.
2. de Pater, S., B.J.P.M. Klemann, and P.J.J. Hooykaas, *True gene-targeting events by CRISPR/Cas-induced DSB repair of the PPO locus with an ectopically integrated repair template.* Scientific Reports, 2018. **8**(1): p. 3338.

3. Wang, M., et al., *Gene Targeting by Homology-Directed Repair in Rice Using a Geminivirus-Based CRISPR/Cas9 System.* Mol Plant, 2017. **10**(7): p. 1007-1010.
4. Miki, D., et al., *CRISPR/Cas9-mediated gene targeting in Arabidopsis using sequential transformation.* Nature Communications, 2018. **9**(1): p. 1967.
5. Roberts, B., et al., *Systematic gene tagging using CRISPR/Cas9 in human stem cells to illuminate cell organization.* Molecular Biology of the Cell, 2017. **28**(21): p. 2854-2874.
6. Begemann, M.B., et al., *Precise insertion and guided editing of higher plant genomes using Cpf1 CRISPR nucleases.* Scientific reports, 2017. **7**(1): p. 11606-11606.
7. Li, C., et al., *Altered levels of histone deacetylase OsHDT1 affect differential gene expression patterns in hybrid rice.* PLoS One, 2011. **6**(7): p. e21789.
