# Supplementary material for: Fusion of the Cas9 endonuclease and the VirD2 relaxase facilitates homology-directed repair for precise genome engineering in rice
Source: Commun Biol. 2020 Jan 23;3:44. doi: 10.1038/s42003-020-0768-9 (PMC6978410; doi:10.1038/s42003-020-0768-9)
Supplement: Supplementary file 2 — Reporting Summary [file 42003_2020_768_MOESM2_ESM.pdf]

nature research

Corresponding author(s): Magdy Mahfouz

Last updated by author(s): Dec 13, 2019

Reporting Summary

Nature Research wishes to improve the reproducibility of the work that we publish. This form provides structure for consistency and transparency in reporting. For further information on Nature Research policies, see Authors & Referees and the Editorial Policy Checklist.

Statistics

For all statistical analyses, confirm that the following items are present in the figure legend, table legend, main text, or Methods section.

n/a	Confirmed	
☐	☒	The exact sample size (*n*) for each experimental group/condition, given as a discrete number and unit of measurement
☐	☒	A statement on whether measurements were taken from distinct samples or whether the same sample was measured repeatedly
☒	☐	The statistical test(s) used AND whether they are one- or two-sided<br>*Only common tests should be described solely by name; describe more complex techniques in the Methods section.*
☒	☐	A description of all covariates tested
☒	☐	A description of any assumptions or corrections, such as tests of normality and adjustment for multiple comparisons
☒	☐	A full description of the statistical parameters including central tendency (e.g. means) or other basic estimates (e.g. regression coefficient) AND variation (e.g. standard deviation) or associated estimates of uncertainty (e.g. confidence intervals)
☒	☐	For null hypothesis testing, the test statistic (e.g. *F*, *t*, *r*) with confidence intervals, effect sizes, degrees of freedom and *P* value noted<br>*Give P values as exact values whenever suitable.*
☒	☐	For Bayesian analysis, information on the choice of priors and Markov chain Monte Carlo settings
☒	☐	For hierarchical and complex designs, identification of the appropriate level for tests and full reporting of outcomes
☒	☐	Estimates of effect sizes (e.g. Cohen's *d*, Pearson's *r*), indicating how they were calculated

Our web collection on statistics for biologists contains articles on many of the points above.

Software and code

Policy information about availability of computer code

Data collection	Sanger sequencing is used to analyze the targeted repair. SnapGene was used to align and analyzed the sequence.
Data analysis	All the genes sequence data were obtained from MSU Rice database "MSU Rice Genome Annotation Project" SnapGene was used to analyze the alignment and sequences. For deep sequencing the true seq platform was used, and the data was analyzed commercially by Life and Soft using CRISPRseq software

For manuscripts utilizing custom algorithms or software that are central to the research but not yet described in published literature, software must be made available to editors/reviewers. We strongly encourage code deposition in a community repository (e.g. GitHub). See the Nature Research guidelines for submitting code & software for further information.

Data

Policy information about availability of data

All manuscripts must include a data availability statement. This statement should provide the following information, where applicable:

- Accession codes, unique identifiers, or web links for publicly available datasets
- A list of figures that have associated raw data
- A description of any restrictions on data availability

The authors declare that all data supporting the finding of this study are available in the manuscript and supplementary information, and are also available from the corresponding author on request.

Field-specific reporting

Please select the one below that is the best fit for your research. If you are not sure, read the appropriate sections before making your selection.

☒ Life sciences ☐ Behavioural & social sciences ☐ Ecological, evolutionary & environmental sciences

For a reference copy of the document with all sections, see nature.com/documents/nr-reporting-summary-flat.pdf

Life sciences study design

All studies must disclose on these points even when the disclosure is negative.

Sample size	In all experiments sample size were selected based on established and published protocols for such experiments. All the experiments were performed with more than three biological repeats and all biological repeats include more than three replicates. According to the experimental results described in the methods, this sample size is sufficient.
Data exclusions	No data is excluded
Replication	All experimental attempts for replication were successful and more than three biological replicates were included in each set of experiments.
Randomization	All the experiments, including the herbicide resistance were randomly place to avoid any location effects.
Blinding	Samples were processed through the standard protocols and in some cases with automated procedures of DNA isolation and sequencing that that results in unbiased outcomes.

Reporting for specific materials, systems and methods

We require information from authors about some types of materials, experimental systems and methods used in many studies. Here, indicate whether each material, system or method listed is relevant to your study. If you are not sure if a list item applies to your research, read the appropriate section before selecting a response.

Materials & experimental systems

n/a	Involved in the study	
☐	☒	Antibodies
☒	☐	Eukaryotic cell lines
☒	☐	Palaeontology
☒	☐	Animals and other organisms
☒	☐	Human research participants
☒	☐	Clinical data

Methods

n/a	Involved in the study	
☒	☐	ChIP-seq
☒	☐	Flow cytometry
☒	☐	MRI-based neuroimaging

Antibodies

Antibodies used	anti-Flag-M2 (Sigma) and anti-Mouse IgG (Santa Cruz biotechnology)
Validation	The anti-Flag M2 was used for CO-IP and Immunoblot was developed with Pierce ECL Western blotting Substrate ThermoFisher Scientific
